# Trends in lipid profile and lipid control among survivors of stroke or myocardial infarction among US adults, 2001–2018

**DOI:** 10.3389/fendo.2023.1128878

**Published:** 2023-03-08

**Authors:** Weiwei Dong, Zhiyong Yang

**Affiliations:** ^1^ Department of Neurosurgery, Shengjing Hospital of China Medical University, Shenyang, China; ^2^ Department of Cardiology, Shengjing Hospital of China Medical University, Shenyang, China

**Keywords:** trend, lipid, stroke, myocardial infarction, NHANES

## Abstract

**Background:**

We aim to analyze the change in lipid profile and lipid control among survivors of stroke and/or myocardial infarction among US adults from 2001–2018.

**Methods:**

In total, 3,736 survivors of stroke and/or myocardial infarction from the 2001–2018 National Health and Nutrition Examination Surveys were included in this study, representing a weighted total population of 110,005,898. Trends for lipid concentration and lipid control rate over time were detected *via* general linear regression analysis and lipid control was compared by sex and race *via* survey-weighted logistic regression analysis.

**Results:**

The total cholesterol, LDL, and triglyceride concentrations were significantly decreased in survivors from the 2001–2002 survey cycle to the 2017–2018 survey cycle (p for trend < 0.01). Lipid control was defined as total cholesterol < 200 mg/dL. Among survivors, the lipid control rate increased from 56.2% (95% CI: 43.9%, 67.7%) in the 2001–2002 survey cycle to 73.2% (95% CI: 64.8%, 80.2%) in the 2017–2018 survey cycle (p for trend < 0.01). Women had a higher lipid concentration and were more likely have poor lipid control compared to men. Non-Hispanic White survivors possessed better lipid control than other races survivors.

**Conclusions:**

Lipid concentrations decreased and lipid control improved in stroke and/or myocardial infarction survivors from 2001 to 2018, with heterogeneity observed according to sex and race.

## 1 Introduction

Cardio-cerebrovascular disease remains the leading cause of mortality and disability in the U.S. population (1, 2). Survivors of cardio-cerebrovascular disease are at increased risk of recurrent cardio-cerebrovascular events, with 28% of all strokes and coronary events combined being recurrent events (2–4). Dyslipidemia is a risk factor for the occurrence and recurrence of cardio-cerebrovascular disease (5, 6). The prevalence of dyslipidemia is increasing due to unhealthy diet and lifestyle (7, 8). Lipid concentration and lipid control trends have been studied in the general population (9), but not in survivors of cardio-cerebrovascular disease. For survivors of stroke and/or myocardial infarction, experts recommend pharmacological interventions to reduce lipid level to prevent recurrence and prolong survival of the survivors (10–12). Although the benefits of dyslipidemia treatment in survivors of stroke and/or myocardial infarction are clear (13), further information is needed regarding the treatment adequacy. Trends in lipid level and lipid control in survivors of stroke and/or myocardial infarction may provide an important reference for recurrence control and prevention, as well as directions for improved interventions in the future.

Therefore, we investigated the changes in lipid level and control, and explored their variation by sex and race in survivors of stroke and/or myocardial infarction among US adults from 2001–2018.

## 2 Material and methods

### 2.1 Study population

The National Health and Nutrition Examination Surveys (NHANES) database was established by the Centers for Disease Control to assess the nutritional and health status of the U.S. population. The survey is conducted every 2 years using a complex stratified multi-step sample to investigate the health status of the entire U.S. population. The NHANES investigators collected data, including demographic data, examination data, laboratory data, and questionnaire data, from in-home interviews and study visits conducted in the mobile examination center. We included survivors of stroke and/or myocardial infarction (n = 3,736, weighted total population of 110,005,898) aged ≥ 20 years in NHANES from 2001 to 2018. Participants who answered “yes” to the question “Has a doctor ever told you that you had a heart attack?” and/or “Has a doctor ever told you that you had a stroke?” were defined as survivors of myocardial infarction and stroke, respectively (14, 15). The survivors’ data included age, race, sex, education level, marital status, poverty to income ratio (PIR), body mass index (BMI), medication use for dyslipidemia, and smoking status. Because of the small sample size for races other than non-Hispanic White, we divided the races into two groups: non-Hispanic White and other races, including Mexican American and non-Hispanic Black. BMI was calculated by dividing the weight (kg) by the square of their height (m^2^). The answer to the use of lipid-lowering medications “yes” or “no”, was based on respondents’ self-report. Smoking status was considered “yes” if participants had smoked ≥ 100 cigarettes in a lifetime (16, 17).

### 2.2 Outcomes

The main outcome included lipid levels (total cholesterol, high-density lipoprotein [HDL], low-density lipoprotein [LDL], and triglycerides), which were examined *via* laboratory measurement. Further information on NHANES laboratory measurement methods and procedures can be found at http://www.cdc.gov/nchs/nhanes/survey_methods.htm. The secondary outcome was lipid control, which was defined as total cholesterol < 200 mg/dL.

### 2.3 Statistical analysis

Statistical analyses were conducted in accordance with the NHANES analysis recommendations. Considering the complex multi-stage survey design of NHANES, we assigned sample weights and stratified and clustered each participant to calculate the national representative estimate. Categorical variables are presented as survey-weighted percentage (95% confidence interval [CI]) and continuous variables are presented as survey-weighted mean (95% CI). Kolmogorov-Smirnov method was utilized to assess data normality. The triglyceride concentration was log-transformed because the distribution was skewed. We evaluated the trends in lipid concentration and lipid control rate over time using a general linear regression analysis, and cross-sectionally analyzed change in lipid control (yes or no) across sex and race subgroups for 4-year survey periods at various times (2001–2004, 2005–2008, 2009–2012, and 2013–2016). Survey cycles were combined to obtain more reliable estimates. To compare cross-sectional lipid control (yes or no) estimates by sex and race, we utilized survey-weighted logistic regression analysis and calculated odds ratio (ORs). All analyses were performed using Empower software (www.empowerstats.com; X&Y solutions, Inc., Boston, MA, USA) and R version 3.4.3 (http://www.Rproject.org, The R Foundation). P-values < 0.05 were considered statistically significant.

## 3 Results

A total of 3,736 participants, who responded with history of stroke and/or myocardial infarction, were included in this study, representing a weighted total population of 110,005,898. The mean age of the survivors was 65.0 years (95% CI: 64.4, 65.6), 54% survivors were male, 74.1% survivors were Non-Hispanic White, and 25.9% participants were other races. The rate of survivors who self-reported taking medications for dyslipidemia increased gradually from 36.1% (95% CI: 30.7%, 41.9%; 2001–2002 survey cycle) to 57.1% (95% CI: 47.4%, 66.2%; 2017–2018 survey cycle). The weighted baseline characteristic of all survivors of stroke and/or myocardial infarction from 2001–2018 is presented in **Table 1**.

**Table 1:** Baseline of characteristic of all surviviors of stroke and or myocardial infarction from 2001-2018, weighted.

Variables	Total	2001-2002	2003-2004	2005-2006	2007-2008	2009-2010	2011-2012	2013-2014	2015-2016	2017-2018
Weighted sample size	110005898	9980317	10705869	12181600	12750840	11213789	12235852	12830109	12962766	15144756
Un-weighted sample size	3736	407	372	363	487	439	386	389	418	475
Age,y^a^	65.0 (64.4,65.6)	65.0 (62.8,67.2)	64.6 (61.9,67.2)	65.8 (63.9,67.6)	65.1 (63.4,66.8)	64.0 (61.9,66.1)	65.1 (63.6,66.7)	65.8 (64.4,67.3)	64.6 (63.1,66.1)	65.0 (63.4,66.7)
Sex										
Man	54.0 (51.6,56.3)	52.8 (47.2,58.3)	51.5 (44.0,58.9)	50.6 (43.1,58.0)	51.8 (45.5,58.0)	59.9 (54.3,65.3)	52.2 (45.2,59.1)	52.1 (43.4,60.7)	58.4 (52.8,63.7)	56.1 (45.7,66.0)
Women	46.0 (43.7,48.4)	47.2 (41.7,52.8)	48.5 (41.1,56.0)	49.4 (42.0,56.9)	48.2 (42.0,54.5)	40.1 (34.7,45.7)	47.8 (40.9,54.8)	47.9 (39.3,56.6)	41.6 (36.3,47.2)	43.9 (34.0,54.3)
Race										
Non-Hispanic White	74.1 (71.4,76.6)	78.3 (71.2,84.0)	80.7 (72.4,87.0)	80.8 (72.5,87.1)	75.1 (64.3,83.4)	73.9 (65.5,81.0)	70.1 (61.6,77.3)	75.3 (67.8,81.5)	64.8 (54.3,74.0)	70.7 (62.2,78.0)
Other races	25.9 (23.4,28.6)	21.7 (16.0,28.8)	19.3 (13.0,27.6)	19.2 (12.9,27.5)	24.9 (16.6,35.7)	26.1 (19.0,34.5)	29.9 (22.7,38.4)	24.7 (18.5,32.2)	35.2 (26.0,45.7)	29.3 (22.0,37.8)
Educational level										
<high school	27.4 (25.3,29.6)	37.6 (31.4,44.1)	30.4 (22.9,39.0)	30.5 (23.8,38.1)	32.6 (27.2,38.5)	30.0 (24.1,36.6)	27.4 (20.5,35.5)	24.5 (18.7,31.3)	22.7 (16.8,30.0)	16.6 (10.5,25.2)
high school	28.7 (26.7,30.9)	25.2 (19.4,32.2)	26.7 (22.1,31.8)	28.7 (21.2,37.8)	29.3 (23.5,36.0)	27.6 (20.9,35.6)	27.3 (19.7,36.6)	28.1 (22.7,34.1)	27.2 (21.9,33.2)	35.9 (30.5,41.6)
>high school	43.8 (41.4,46.3)	37.2 (31.5,43.3)	42.9 (35.8,50.4)	40.8 (30.9,51.4)	38.1 (30.7,46.0)	42.4 (33.6,51.7)	45.3 (38.5,52.3)	47.4 (41.6,53.4)	50.1 (42.6,57.6)	47.5 (39.2,56.0)
Marital status										
Married/Living with partner	58.0 (55.8,60.3)	57.8 (50.4,64.9)	57.9 (47.5,67.6)	57.9 (53.1,62.5)	59.1 (52.7,65.3)	61.3 (54.5,67.7)	57.6 (47.3,67.2)	56.5 (49.8,62.9)	54.7 (49.2,60.0)	59.7 (52.4,66.6)
Widowed/Divorced/Separated	35.2 (33.1,37.3)	36.1 (29.4,43.3)	36.2 (27.2,46.4)	38.1 (33.1,43.3)	33.9 (26.9,41.7)	32.2 (27.3,37.6)	34.9 (28.2,42.3)	35.4 (29.8,41.5)	35.5 (30.3,41.1)	34.4 (27.9,41.4)
Never married	6.8 (5.8,7.9)	6.1 (3.8,9.6)	5.9 (3.2,10.6)	4.0 (2.9,5.6)	7.0 (3.9,12.2)	6.4 (3.7,11.1)	7.5 (3.9,14.0)	8.1 (5.7,11.4)	9.8 (7.1,13.4)	6.0 (3.9,9.0)
PIR										
<1	16.0 (14.4,17.8)	13.5 (9.5,18.7)	12.7 (9.6,16.5)	15.2 (11.0,20.7)	17.7 (13.0,23.7)	17.7 (13.3,23.1)	20.6 (15.8,26.3)	17.7 (11.0,27.4)	16.2 (10.7,24.0)	12.9 (10.0,16.3)
1-2	27.8 (25.8,29.9)	28.9 (22.1,36.8)	33.2 (26.2,40.9)	25.2 (18.6,33.0)	28.1 (25.4,30.9)	27.0 (21.5,33.3)	24.4 (17.7,32.8)	32.1 (24.3,41.1)	30.8 (23.7,38.8)	22.2 (18.1,26.9)
2-3	16.0 (14.1,18.0)	15.1 (9.2,23.7)	16.9 (12.6,22.3)	21.7 (14.8,30.7)	15.7 (11.6,21.0)	12.6 (8.2,18.8)	16.3 (10.1,25.4)	15.7 (11.4,21.2)	12.1 (6.6,21.2)	17.3 (13.0,22.5)
3-4	9.5 (8.1,11.1)	8.9 (5.8,13.3)	9.7 (4.6,19.4)	11.7 (7.6,17.6)	5.7 (3.7,8.5)	13.8 (10.7,17.8)	7.5 (4.1,13.3)	10.5 (6.6,16.3)	11.7 (6.4,20.5)	7.1 (4.7,10.7)
4-5	7.0 (5.7,8.5)	9.1 (5.0,16.0)	9.5 (5.0,17.4)	6.3 (4.1,9.5)	5.7 (3.2,10.0)	6.1 (4.0,9.1)	9.0 (5.1,15.3)	6.6 (3.2,13.1)	7.4 (3.1,16.5)	4.5 (2.2,9.0)
≥5	15.3 (13.5,17.4)	17.1 (11.0,25.8)	13.1 (8.8,18.9)	13.2 (8.9,19.1)	17.5 (12.0,24.7)	17.2 (10.4,27.2)	13.6 (9.1,19.8)	12.1 (8.2,17.6)	11.4 (7.0,18.0)	21.9 (15.6,29.8)
Unknown	8.3 (7.1,9.7)	7.5 (5.1,10.8)	5.0 (2.6,9.2)	6.7 (3.4,12.8)	9.7 (7.5,12.4)	5.6 (3.6,8.6)	8.5 (5.4,13.2)	5.3 (3.3,8.3)	10.4 (6.6,16.1)	14.2 (8.9,21.9)
BMI										
<25kg/m^2^	20.1 (18.7,21.6)	15.7 (11.9,20.6)	24.9 (19.0,31.8)	18.1 (14.9,21.8)	22.4 (18.4,27.0)	21.0 (17.9,24.5)	22.3 (18.2,27.1)	23.5 (18.4,29.6)	19.1 (15.2,23.7)	15.0 (11.4,19.5)
25 to 30kg/m^2^	29.6 (27.6,31.7)	28.9 (21.4,37.7)	32.7 (26.3,39.9)	33.7 (27.9,40.1)	31.2 (27.1,35.6)	27.4 (23.1,32.2)	27.0 (20.7,34.2)	30.4 (24.6,36.9)	27.4 (22.1,33.5)	28.0 (20.4,37.1)
≥30kg/m^2^	40.5 (38.4,42.7)	29.3 (21.5,38.6)	33.8 (29.2,38.7)	39.0 (33.7,44.7)	37.9 (32.6,43.6)	47.5 (43.2,51.8)	42.0 (34.1,50.2)	39.8 (31.8,48.4)	46.3 (39.4,53.2)	45.5 (38.2,52.9)
Unknown	9.7 (8.5,11.1)	26.1 (19.2,34.4)	8.6 (5.8,12.4)	9.1 (5.4,14.9)	8.4 (5.4,13.0)	4.1 (2.9,6.0)	8.7 (5.8,12.9)	6.2 (3.6,10.5)	7.2 (3.6,14.1)	11.6 (8.3,15.8)
Take medication for dyslipidemia										
Yes	48.6 (46.2,51.0)	36.1 (30.7,41.9)	35.5 (29.2,42.4)	42.3 (35.2,49.7)	43.0 (38.3,47.8)	41.7 (35.3,48.4)	60.2 (51.4,68.4)	57.2 (51.4,62.7)	56.8 (47.7,65.4)	57.1 (47.4,66.2)
No	7.0 (6.0,8.2)	4.7 (2.8,7.6)	6.5 (2.8,14.0)	2.7 (1.3,5.5)	7.7 (5.5,10.6)	4.6 (2.8,7.5)	5.6 (3.6,8.7)	6.3 (4.2,9.3)	8.5 (5.1,13.8)	14.3 (10.6,19.1)
Unknown	44.4 (42.1,46.7)	59.2 (52.2,65.9)	58.0 (49.4,66.2)	55.0 (47.0,62.8)	49.3 (44.6,54.1)	53.7 (47.1,60.2)	34.1 (26.5,42.7)	36.5 (31.0,42.4)	34.7 (28.5,41.5)	28.6 (22.2,36.1)
Smoke										
Yes	63.2 (60.9,65.4)	64.6 (56.7,71.8)	64.5 (56.2,72.0)	67.9 (60.2,74.8)	60.8 (54.7,66.7)	59.5 (53.8,65.1)	63.2 (54.4,71.1)	63.9 (59.1,68.4)	64.2 (55.0,72.4)	60.7 (53.8,67.2)
No	36.8 (34.6,39.1)	35.4 (28.2,43.3)	35.5 (28.0,43.8)	32.1 (25.2,39.8)	39.2 (33.3,45.3)	40.5 (34.9,46.2)	36.8 (28.9,45.6)	36.1 (31.6,40.9)	35.8 (27.6,45.0)	39.3 (32.8,46.2)

PIR, poverty to income ratio; BMI, body mass index.

a Continuous variable presented as survey-weighted mean (95% CI).

Categorical variables were presented as survey-weighted percentage (95% CI).

The survey weighting method occasionally produced estimates in decimal numbers. The sum of the numbers may not add up to the heading totals when they are rounded and added.

### 3.1 Trends in lipid concentration in the US adult population with a history of stroke and/or myocardial infarction

**Figure 1:**
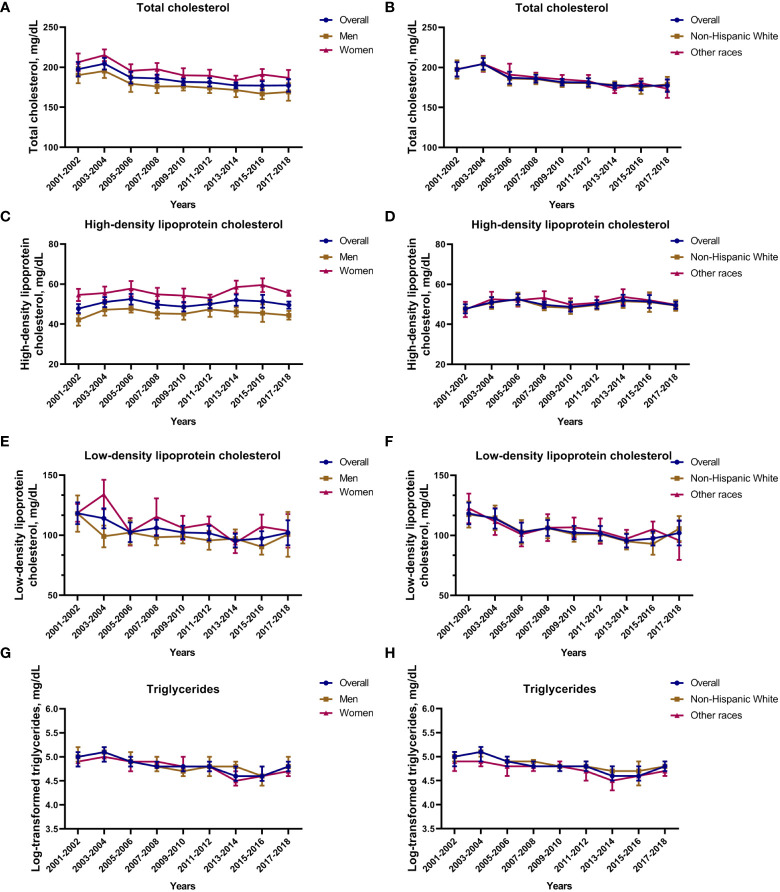
Total cholesterol, HDL, LDL and triglyceride concertation among survivors of stroke and/or myocardial infarction. **(A)** Total cholesterol concentration based on sex; **(B)** total cholesterol concentration based on race; **(C)** HDD concentration based on sex; **(D)** HDD concentration based on race; **(E)** LDD concentration based on sex; **(F)** LDD concentration based on race; **(G)** log-transformed triglyceride concentration based on sex; **(H)** log-transformed triglyceride concentration based on race. Nationally representative estimates of the survivors of stroke and/or myocardial infarction aged ≥ 20 years in NHANES from 2001 to 2018 (total cholesterol n=3,245, representing a weighted total population of 97,726,894; HDL n=3,244, representing a weighted total population of 97,706,175; LDL n=1,552, representing a weighted total population of 47,384,996; triglyceride n=1,605, representing a weighted total population of 48,694,152). Estimates are presented as survey-weighted mean and 95% confidence intervals.

Because fasting blood tests were not performed in some survivors, our trend analysis of lipid concentrations (total cholesterol, HDL, LDL, and triglycerides) included only those survivors who reported fasting total cholesterol, HDL, LDL, and triglyceride values accordingly. The weighted baseline characteristics of survivors with fasting lipid value of total cholesterol (n=3,245, representing a weighted total population of 97,726,894), HDL(n=3,244, representing a weighted total population of 97,706,175), LDL(n=1,552, representing a weighted total population of 47,384,996), and triglyceride (n=1,605, representing a weighted total population of 48,694,152) are shown in **Tables S1–S4**. Among survivors of stroke and/or myocardial infarction, total cholesterol was significantly decreased from 197.5 mg/dL (95% CI: 188.5, 206.5 mg/dL) in the 2001–2002 survey cycle to 177.3 mg/dL (95% CI: 169.4, 185.2 mg/dL) in the 2017–2018 survey cycle (p for trend < 0.01). We next analyzed the heterogeneity of the total cholesterol level according to sex and race. The results showed that total cholesterol was higher among female survivors than male survivors. The decreased trend in total cholesterol was observed in both male and female survivors, while no obvious decreased trend in heterogeneity was observed according to race (**Figures 1A, B**). Moreover, we found no significant change trend in HDL level among survivors. Although HDL was higher among female survivors than male survivors, no significant difference was observed between non-Hispanic White and other races (**Figures 1C, D**). LDL was significantly decreased from 118.2 mg/dL (95% CI: 109.2, 127.3 mg/dL) in the 2001–2002 survey cycle to 102 mg/dL (95% CI: 91.6, 112.4 mg/dL) in the 2017–2018 survey cycle (p for trend < 0.01). The sex and race subgroup had a similar decreased trend in LDL level. LDL was higher among women survivors than men survivors, but no significant difference was observed between non-Hispanic White and other races (**Figures 1E, F**). In terms of triglycerides, the log-transformed triglycerides level was decreased from 5.0 mg/dL (95% CI: 4.8, 5.1 mg/dL) in the 2001–2002 survey cycle to 4.8 mg/dL (95% CI: 4.7, 4.9 mg/dL) in the 2017–2018 survey cycle (p for trend < 0.01). The sex and race subgroup had a similarly decreased trend in triglycerides level, and no significant heterogeneity was observed by sex and race (**Figures 1G, H**). Further, covariates (age, gender, race, educational level, marital status, PIR, BMI, taking lipid-lowering drugs, and smoke) were included and general linear regression analysis was performed to investigate trends of change in lipid concentrations of total cholesterol, HDL, LDL, and triglycerides. The results were consistent after adjusting for covariates. In summary, with the exception of HDL, the levels of lipid (total cholesterol, LDL, and triglycerides) showed a decreasing trend.

### 3.2 Trends in lipid control rate in the US adult population with a history of stroke and/or myocardial infarction

**Figure 2:**
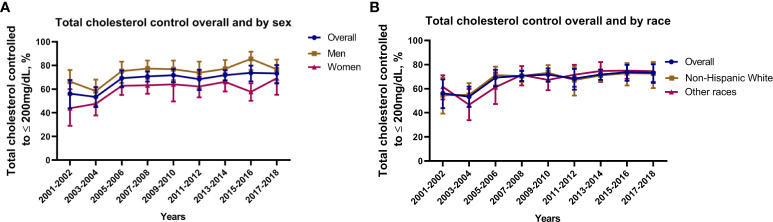
Lipid control rate among survivors of stroke and/or myocardial infarction. **(A)** Lipid control rate based on sex; **(B)** Lipid control rate based on race. Nationally representative estimates of the survivors of stroke and/or myocardial infarction aged ≥ 20 years in NHANES from 2001 to 2018 (n=3,245, representing a weighted total population of 97,726,894). Estimates are presented as survey-weighted percentage and 95% confidence intervals.

Among survivors of stroke and/or myocardial infarction, the lipid control rate increased from 56.2% (95% CI: 43.9%, 67.7%) in the 2001–2002 survey cycle to 73.2% (95% CI: 64.8%, 80.2%) in the 2017–2018 survey cycle (p for trend < 0.01). When adjusted for covariates mentioned above, we also observed increased trend in lipid control among survivors. The sex and race subgroup had a similarly increased trend in lipid control rate. Lipid control rate was higher among male survivors than females, but no significant difference was observed between non-Hispanic White and other races (**Figure 2**).

### 3.3 Comparison of lipid control based on sex and race among survivors of stroke and/or myocardial infarction

**Figure 3:**
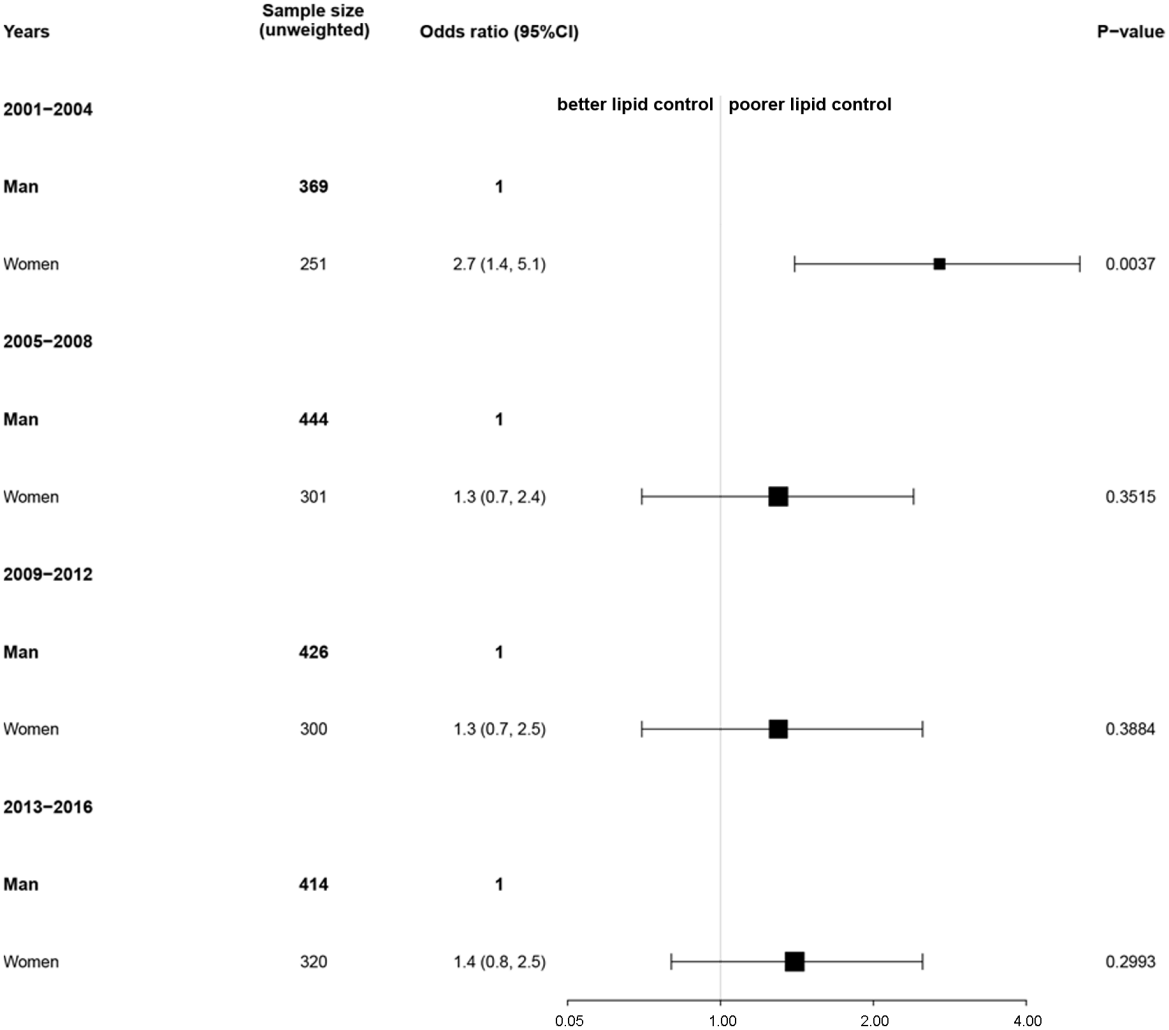
Association of sex with lipid control among survivors of stroke and/or myocardial infarction. Nationally representative estimates of the survivors of stroke and/or myocardial infarction aged ≥ 20 years in NHANES from 2001 to 2016 (n=3,245, representing a weighted total population of 97,726,894). Poor or good lipid control was defined as total cholesterol > 200 mg/dL or not, respectively. The reference group was men. An odds ratio > 1 indicates a higher rate of poor lipid control.

**Figure 4:**
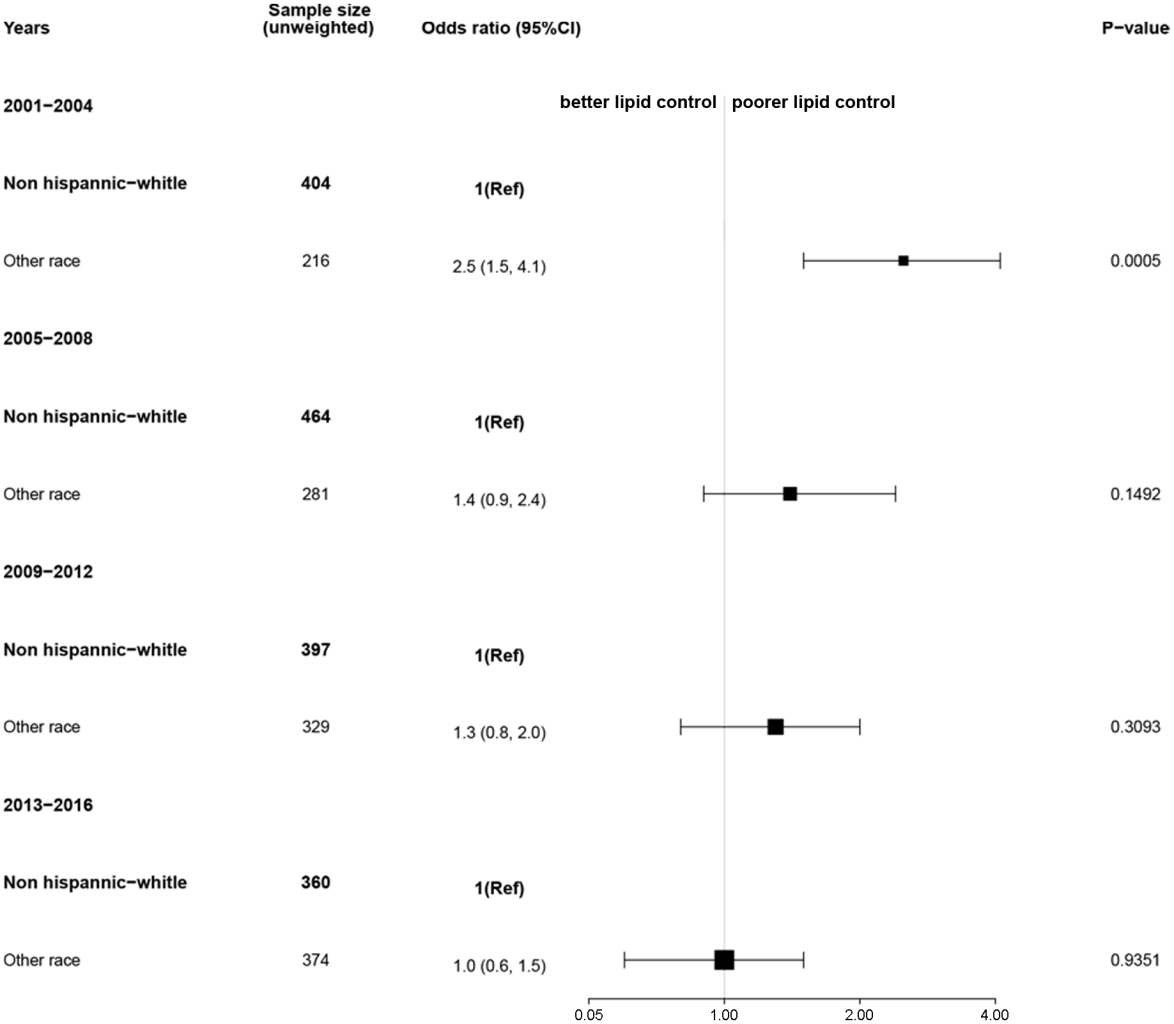
Association of race with lipid control among survivors of stroke and/or myocardial infarction. Nationally representative estimates of the survivors of stroke and/or myocardial infarction aged ≥ 20 years in NHANES from 2001 to 2016 (n=3,245, representing a weighted total population of 97,726,894). Poor or good lipid control was defined as total cholesterol > 200 mg/dL or not, respectively. The reference group was non-Hispanic White race. An odds ratio > 1 indicates a higher rate of poor lipid control.

Given that there was a difference in the lipid profile by sex, we next conducted a comparison of lipid control by sex and race. In 2001–2004, the poor lipid control (total cholesterol > 200 mg/dL) was significantly higher for female compared to male survivors (OR = 2.7, 95% CI: [1.4, 5.1]). Similar results were observed in 2005–2008 (OR = 1.3, 95% CI: [0.7, 2.4]), 2009–2012 (OR = 1.3, 95% CI: [0.7, 2.5]), and 2013–2016 (OR = 1.4, 95% CI: [0.8, 2.5]). Overall, female survivors of stroke and/or myocardial infarction were more likely have poor lipid control compared to males (**Figure 3**). We also conducted a comparison of lipid control by race. In 2001–2004, when compared to non-Hispanic White, the poor lipid control was significantly higher for other races (OR = 2.5, 95% CI: [1.5, 4.1]). The OR value showed a gradual decrease over time. In 2005–2008, 2009–2012, and 2013–2016, the ORs were 1.4, 95% CI: (0.9, 2.4), 1.3, 95% CI: (0.8, 2.0), and 1.0, 95% CI: (0.6, 1.5), respectively. The difference in poor lipid control between non-Hispanic White and other races among survivors of stroke and/or myocardial infarction decreased gradually over time (**Figure 4**).

## 4 Discussion

In this study, we explored the lipid profile and control among survivors of stroke and/or myocardial infarction. Our results showed that the proportion of survivors taking medication to control dyslipidemia increased, the total cholesterol, triglycerides, and LDL levels, showed a decreasing trend from 2001 to 2018, and lipid control increased among survivors of stroke and/or myocardial infarction. We also found that men had better lipid control compared to female survivors, and non-Hispanic White survivors had better lipid control than other races; however, the difference in lipid control diminished gradually in individuals of non-Hispanic White race compared to other races.

The national trend of better control of dyslipidemia in survivors of stroke and/or myocardial infarction may be related to the guidance of the new guideline. The 2018 American Heart Association (AHA)/American College of Cardiology (ACC) Multisociety Guideline recommends further strengthening the management of blood cholesterol and high-intensity statin therapy for high-risk patients. Patients with atherosclerotic cardiovascular disease should achieve a ≥ 50% reduction in LDL-cholesterol (LDL-C) level, as a primary goal of therapy (18). For adults aged 40 to 75 years with one or more cardiovascular disease risk factor and an estimated 10-year cardiovascular disease risk of ≥ 10%, the US Preventive Services Task Force suggests that clinicians prescribe a statin for the primary prevention (19). A study has revealed that high dose statin therapy reduced cardiovascular morbidity but not mortality compared to low or moderate dose statin therapy (20). Moreover, a systematic review for the US Preventive Services Task Force reported that statin therapy was correlated with a decreased risk of cardiovascular events and all-cause cardiovascular mortality and all-cause mortality (risk ratio [RR], 0.86 [95% CI, 0.80 to 0.93]; stroke (RR, 0.71 [95% CI, 0.62 to 0.82]); myocardial infarction (RR, 0.64 [95% CI, 0.57 to 0.71]) (21). However, high dose statin therapy has been found to be correlated with adverse complications such as myopathy and incident diabetes (22). The Synopsis of the 2020 Updated U.S. Department of Veterans Affairs and U.S. Department of Defense Clinical Practice Guideline recommended moderate intensity statin treatment as the foundation of pharmacologic treatment for the secondary prevention for cardiovascular disease (23).

In this study, we found that female survivors of stroke and/or myocardial infarction had high lipid levels and poorer lipid control than males. Similarly, a Chinese study of 1484 acute ischemic stroke in elderly patients (>75 years) from 2005 to 2013, found that women had higher levels of TC, TG, HDL-C, and LDL-C, and were more likely to have dyslipidemia compared to men (24). In the general population, the total cholesterol level decreased more in males than females from 2001 to 2016 in the US. The average total cholesterol levels for males and females from 2001 to 2004 and 2013 to 2016 were 201 and 188 mg/dL, respectively. Sex differences were also found in dyslipidemia control, with control rates of 51% for women and 63% for men from 2013 to 2016 in the US (25). Statin efficacy is closely related to medication adherence, and studies have found that female sex was associated with lower statin adherence (26). Female’s higher rates of visceral fat increase with age compared to males, which may explain the higher dyslipidemia rate among female survivors of stroke and/or myocardial infarction (27). Studies have found the with increasing age, females are more sedentary than males and are more likely to develop mobility impairments (28, 29). Overall, differences in healthy lifestyle, medication adherence, and physical function contribute to differences in lipid levels and lipid control between female and male survivors of stroke and/or myocardial infarction. Differences in genetic factors, lifestyle, dietary habits, socioeconomic status, and medical resources may explain the heterogeneity of lipid levels and lipid control in survivors of stroke and/or myocardial infarction by race. In this study, we found that the difference in lipid control diminished gradually between non-Hispanic White races compared to other races.

Our study has several limitations. First, lipid parameters were missing in some of the survivors, trend analysis was performed only among survivors with lipid parameters, and we cannot excluded the presence of selective bias that could affect our results. Second, because the small sample size of survivors of other races in this study, we only divided race into non-Hispanic White and other races. Future studies with larger sample sizes may detect differences in lipid levels and lipid control among survivors of different races. Third, the definition of lipid control varies across guidelines, and the definition of lipid control chosen for this study is consistent with that in previous reports (30). Fourth, when analyzing lipid control based on sex and race, we combined survey cycles at 4-year survey periods to cross-sectionally analyze changes in lipid control across subgroups, and data of the 2017–2018 survey cycle were not included in the analysis. Finally, it is unclear whether the results of this study will continue to be consistent until the 2021–2022 survey cycle, especially under the impact of the new coronary pneumonia epidemic.

## 5 Conclusion

In this cross-sectional study, we observed that lipid concentrations decreased in stroke and/or myocardial infarction survivors. Survivors had improved lipid control, however, there was heterogeneity based on sex and race.

## Data availability statement

Publicly available datasets were analyzed in this study. This data can be found here: https://wwwn.cdc.gov/nchs/nhanes/Default.aspx.

## Author contributions

WD: Conceptualization, Methodology, Software, Investigation, Data curation, Writing-original draft. ZY: Visualization, Supervision, Writing-review & editing. All authors contributed to the article and approved the submitted version.
