# Supplementary material for: Trends in lipid profile and lipid control among survivors of stroke or myocardial infarction among US adults, 2001–2018
Source: Front Endocrinol (Lausanne). 2023 Mar 8;14:1128878. doi: 10.3389/fendo.2023.1128878 (PMC10031105; doi:10.3389/fendo.2023.1128878)
Supplement: Supplementary file 1 [file Table_1.docx]

Table S1 Weighted demographic characteristics of survivors for trend analysis of cholesterol

| Variables | 2001-2002 | 2003-2004 | 2005-2006 | 2007-2008 | 2009-2010 | 2011-2012 | 2013-2014 | 2015-2016 | 2017-2018 |
| --- | --- | --- | --- | --- | --- | --- | --- | --- | --- |
| Weighted sample size | 7959389 | 9437775 | 10740660 | 11252411 | 10452925 | 10845801 | 12065938 | 11753744 | 13218251 |
| Un-weighted sample size | 303 | 317 | 321 | 424 | 400 | 326 | 358 | 376 | 420 |
| Age,y^a^ | 63.7 (61.6 ,65.8) | 63.6 (60.7 ,66.5) | 65.9 (64.1 ,67.8) | 64.8 (62.9 ,66.6) | 63.8 (61.7 ,65.9) | 65.1 (63.2 ,66.9) | 65.7 (64.1 ,67.2) | 64.1 (62.4 ,65.7) | 64.7 (62.9 ,66.4) |
| Sex |  |  |  |  |  |  |  |  |  |
| Man | 54.5 (48.3 ,60.6) | 53.8 (46.3 ,61.1) | 52.1 (43.6 ,60.4) | 53.7 (46.7 ,60.5) | 60.4 (55.0 ,65.6) | 53.9 (46.6 ,61.0) | 52.5 (43.3 ,61.5) | 57.5 (51.0 ,63.8) | 53.9 (44.8 ,62.8) |
| Women | 45.5 (39.4 ,51.7) | 46.2 (38.9 ,53.7) | 47.9 (39.6 ,56.4) | 46.3 (39.5 ,53.3) | 39.6 (34.4 ,45.0) | 46.1 (39.0 ,53.4) | 47.5 (38.5 ,56.7) | 42.5 (36.2 ,49.0) | 46.1 (37.2 ,55.2) |
| race |  |  |  |  |  |  |  |  |  |
| Non-Hispanic White | 78.0 (70.1 ,84.2) | 81.2 (73.0 ,87.3) | 80.6 (71.6 ,87.2) | 76.2 (66.0 ,84.0) | 75.2 (66.6 ,82.2) | 72.1 (63.8 ,79.2) | 76.6 (69.4 ,82.6) | 64.4 (53.1 ,74.3) | 69.3 (60.3 ,77.1) |
| Other races | 22.0 (15.8 ,29.9) | 18.8 (12.7 ,27.0) | 19.4 (12.8 ,28.4) | 23.8 (16.0 ,34.0) | 24.8 (17.8 ,33.4) | 27.9 (20.8 ,36.2) | 23.4 (17.4 ,30.6) | 35.6 (25.7 ,46.9) | 30.7 (22.9 ,39.7) |
| Educational level |  |  |  |  |  |  |  |  |  |
| <high school | 39.3 (30.3 ,49.1) | 29.5 (21.9 ,38.5) | 29.2 (21.5 ,38.3) | 32.3 (25.7 ,39.7) | 29.4 (23.6 ,36.1) | 25.5 (18.0 ,34.9) | 23.7 (17.6 ,31.3) | 21.1 (16.1 ,27.1) | 16.5 (9.8 ,26.5) |
| high school | 25.0 (18.3 ,33.1) | 26.9 (22.3 ,32.0) | 28.5 (20.8 ,37.7) | 29.4 (24.0 ,35.3) | 27.6 (20.7 ,35.6) | 28.0 (19.8 ,37.8) | 28.4 (22.7 ,34.9) | 28.0 (23.4 ,33.1) | 35.1 (28.2 ,42.7) |
| >high school | 35.7 (27.7 ,44.5) | 43.6 (35.3 ,52.3) | 42.3 (31.9 ,53.4) | 38.4 (31.3 ,45.9) | 43.0 (34.6 ,51.8) | 46.5 (38.6 ,54.6) | 47.8 (41.5 ,54.3) | 50.9 (43.9 ,57.8) | 48.3 (39.2 ,57.5) |
| Marital status |  |  |  |  |  |  |  |  |  |
| Married/Living with partner | 60.9 (53.4 ,68.0) | 60.8 (50.5 ,70.2) | 57.3 (52.2 ,62.2) | 61.8 (55.5 ,67.8) | 63.7 (57.5 ,69.4) | 60.7 (51.3 ,69.3) | 57.1 (50.5 ,63.4) | 57.3 (51.3 ,63.0) | 60.6 (53.1 ,67.7) |
| Widowed/ Divorced/ Separated | 32.1 (25.1 ,40.2) | 33.0 (24.2 ,43.2) | 39.2 (33.9 ,44.7) | 31.0 (25.0 ,37.7) | 30.9 (25.9 ,36.4) | 32.2 (26.5 ,38.6) | 35.1 (29.5 ,41.1) | 32.6 (27.4 ,38.3) | 34.4 (28.0 ,41.4) |
| Never married | 6.9 (4.2 ,11.2) | 6.2 (3.4 ,11.1) | 3.5 (2.3 ,5.5) | 7.2 (3.9 ,12.7) | 5.4 (3.4 ,8.6) | 7.1 (3.5 ,14.0) | 7.8 (5.2 ,11.6) | 10.1 (7.1 ,14.2) | 5.0 (3.4 ,7.2) |
| PIR |  |  |  |  |  |  |  |  |  |
| <1 | 14.6 (9.7 ,21.4) | 12.8 (9.0 ,18.0) | 15.2 (10.2 ,22.1) | 17.7 (12.5 ,24.6) | 17.5 (12.7 ,23.5) | 19.4 (14.5 ,25.6) | 17.5 (10.7 ,27.4) | 16.4 (10.6 ,24.5) | 13.8 (10.7 ,17.6) |
| 1-2 | 28.4 (21.0 ,37.3) | 32.2 (25.3 ,39.9) | 24.6 (17.8 ,33.0) | 27.4 (24.4 ,30.7) | 26.4 (20.6 ,33.2) | 26.0 (18.6 ,35.1) | 32.2 (24.1 ,41.6) | 30.7 (24.0 ,38.3) | 23.6 (19.5 ,28.4) |
| 2-3 | 16.1 (9.7 ,25.7) | 17.6 (12.9 ,23.5) | 21.5 (15.0 ,30.0) | 16.3 (11.2 ,23.2) | 11.8 (7.8 ,17.4) | 14.8 (8.6 ,24.4) | 16.0 (11.3 ,22.0) | 12.9 (6.9 ,22.9) | 17.8 (13.1 ,23.6) |
| 3-4 | 9.0 (5.6 ,14.2) | 9.5 (4.4 ,19.5) | 12.9 (8.3 ,19.3) | 6.2 (3.9 ,9.7) | 14.7 (11.3 ,19.0) | 8.4 (4.6 ,14.8) | 10.9 (7.0 ,16.8) | 12.1 (6.5 ,21.5) | 6.4 (4.4 ,9.2) |
| 4-5 | 11.3 (6.2 ,19.6) | 10.1 (5.1 ,19.0) | 4.6 (2.5 ,8.2) | 6.2 (3.4 ,11.0) | 6.4 (4.2 ,9.7) | 9.8 (5.3 ,17.2) | 6.7 (3.2 ,13.5) | 7.6 (3.1 ,17.5) | 4.5 (1.9 ,10.3) |
| ≥5 | 15.8 (9.4 ,25.3) | 13.4 (8.6 ,20.4) | 14.7 (9.9 ,21.2) | 17.6 (12.6 ,24.1) | 18.1 (10.9 ,28.6) | 14.4 (9.4 ,21.4) | 12.3 (8.3 ,17.9) | 11.0 (6.7 ,17.5) | 23.3 (16.6 ,31.8) |
| Unknown | 4.8 (2.5 ,8.8) | 4.4 (2.2 ,8.6) | 6.5 (3.3 ,12.4) | 8.5 (6.3 ,11.5) | 5.0 (3.3 ,7.6) | 7.1 (3.8 ,12.9) | 4.4 (2.8 ,6.9) | 9.3 (5.7 ,14.8) | 10.5 (5.7 ,18.6) |
| BMI |  |  |  |  |  |  |  |  |  |
| <25kg/m^2^ | 17.9 (13.9 ,22.8) | 25.9 (19.5 ,33.5) | 17.6 (13.7 ,22.3) | 24.5 (20.3 ,29.3) | 21.5 (18.5 ,24.8) | 22.7 (17.9 ,28.3) | 24.3 (18.9 ,30.6) | 19.7 (15.6 ,24.5) | 16.4 (12.2 ,21.7) |
| 25 to 30kg/m^2^ | 35.2 (25.7 ,45.9) | 36.2 (29.7 ,43.2) | 35.3 (28.9 ,42.3) | 33.8 (29.0 ,39.0) | 28.1 (23.2 ,33.6) | 28.5 (21.4 ,36.8) | 32.0 (26.2 ,38.4) | 29.3 (24.1 ,35.0) | 31.3 (22.6 ,41.6) |
| ≥30kg/m^2^ | 35.1 (25.7 ,45.7) | 35.1 (30.1 ,40.5) | 43.2 (37.2 ,49.4) | 39.0 (33.1 ,45.2) | 48.6 (43.6 ,53.7) | 44.2 (35.4 ,53.4) | 41.2 (32.7 ,50.2) | 48.0 (41.4 ,54.6) | 49.5 (41.4 ,57.5) |
| Unknown | 11.8 (6.2 ,21.2) | 2.8 (1.7 ,4.6) | 3.9 (2.1 ,7.0) | 2.7 (1.8 ,4.1) | 1.8 (0.8 ,3.9) | 4.6 (2.0 ,10.3) | 2.5 (1.3 ,5.0) | 3.1 (1.2 ,7.8) | 2.9 (1.0 ,7.6) |
| Take medication for dyslipidemia |  |  |  |  |  |  |  |  |  |
| Yes | 38.5 (32.7 ,44.6) | 34.7 (28.4 ,41.6) | 42.9 (36.7 ,49.4) | 43.6 (38.1 ,49.2) | 42.1 (35.3 ,49.3) | 61.6 (52.0 ,70.3) | 57.8 (52.5 ,62.9) | 57.4 (47.6 ,66.6) | 58.4 (49.2 ,67.0) |
| No | 5.2 (3.0 ,8.9) | 7.3 (3.3 ,15.3) | 1.8 (0.9 ,3.3) | 7.3 (5.1 ,10.3) | 4.9 (3.0 ,8.1) | 5.6 (3.3 ,9.2) | 5.9 (4.0 ,8.7) | 8.4 (4.5 ,14.9) | 13.0 (9.0 ,18.3) |
| Unknown | 56.3 (48.6 ,63.7) | 58.0 (49.2 ,66.3) | 55.3 (48.7 ,61.8) | 49.2 (43.6 ,54.8) | 52.9 (45.7 ,60.0) | 32.9 (25.1 ,41.7) | 36.3 (31.0 ,42.0) | 34.2 (27.7 ,41.4) | 28.7 (22.5 ,35.7) |
| Smoke |  |  |  |  |  |  |  |  |  |
| Yes | 67.6 (59.4 ,74.8) | 65.7 (56.7 ,73.6) | 68.5 (60.9 ,75.3) | 62.2 (55.3 ,68.6) | 59.7 (53.9 ,65.2) | 63.7 (54.5 ,72.0) | 64.1 (59.0 ,69.0) | 62.8 (53.1 ,71.7) | 60.7 (53.8 ,67.2) |
| No | 32.4 (25.2 ,40.6) | 34.3 (26.4 ,43.3) | 31.5 (24.7 ,39.1) | 37.8 (31.4 ,44.7) | 40.3 (34.8 ,46.1) | 36.3 (28.0 ,45.5) | 35.9 (31.0 ,41.0) | 37.2 (28.3 ,46.9) | 39.3 (32.8 ,46.2) |

Abbreviation: PIR, poverty to income ratio; BMI, body mass index

^a^ Continuous variable presented as survey-weighted mean (95% CI).

Categorical variables were presented as survey-weighted percentage (95% CI).

The survey weighting method occasionally produced estimates in decimal numbers. The sum of the numbers may not add up to the heading totals when they are rounded and added.

Table S2 Weighted demographic characteristics of survivors for trend analysis of HDL

| Variables | 2001-2002 | 2003-2004 | 2005-2006 | 2007-2008 | 2009-2010 | 2011-2012 | 2013-2014 | 2015-2016 | 2017-2018 |
| --- | --- | --- | --- | --- | --- | --- | --- | --- | --- |
| Weighted sample size | 7959389 | 9417056 | 10740660 | 11252411 | 10452925 | 10845801 | 12065938 | 11753744 | 13218251 |
| Un-weighted sample size | 303 | 316 | 321 | 424 | 400 | 326 | 358 | 376 | 420 |
| Age,y^a^ | 63.7 (61.6 ,65.8) | 63.5 (60.7 ,66.4) | 65.9 (64.1 ,67.8) | 64.8 (62.9 ,66.6) | 63.8 (61.7 ,65.9) | 65.1 (63.2 ,66.9) | 65.7 (64.1 ,67.2) | 64.1 (62.4 ,65.7) | 64.7 (62.9 ,66.4) |
| Sex |  |  |  |  |  |  |  |  |  |
| Man | 54.5 (48.3 ,60.6) | 53.9 (46.5 ,61.1) | 52.1 (43.6 ,60.4) | 53.7 (46.7 ,60.5) | 60.4 (55.0 ,65.6) | 53.9 (46.6 ,61.0) | 52.5 (43.3 ,61.5) | 57.5 (51.0 ,63.8) | 53.9 (44.8 ,62.8) |
| Women | 45.5 (39.4 ,51.7) | 46.1 (38.9 ,53.5) | 47.9 (39.6 ,56.4) | 46.3 (39.5 ,53.3) | 39.6 (34.4 ,45.0) | 46.1 (39.0 ,53.4) | 47.5 (38.5 ,56.7) | 42.5 (36.2 ,49.0) | 46.1 (37.2 ,55.2) |
| race |  |  |  |  |  |  |  |  |  |
| Non-Hispanic White | 78.0 (70.1 ,84.2) | 81.1 (72.9 ,87.3) | 80.6 (71.6 ,87.2) | 76.2 (66.0 ,84.0) | 75.2 (66.6 ,82.2) | 72.1 (63.8 ,79.2) | 76.6 (69.4 ,82.6) | 64.4 (53.1 ,74.3) | 69.3 (60.3 ,77.1) |
| Other races | 22.0 (15.8 ,29.9) | 18.9 (12.7 ,27.1) | 19.4 (12.8 ,28.4) | 23.8 (16.0 ,34.0) | 24.8 (17.8 ,33.4) | 27.9 (20.8 ,36.2) | 23.4 (17.4 ,30.6) | 35.6 (25.7 ,46.9) | 30.7 (22.9 ,39.7) |
| Educational level |  |  |  |  |  |  |  |  |  |
| <high school | 39.3 (30.3 ,49.1) | 29.6 (21.9 ,38.6) | 29.2 (21.5 ,38.3) | 32.3 (25.7 ,39.7) | 29.4 (23.6 ,36.1) | 25.5 (18.0 ,34.9) | 23.7 (17.6 ,31.3) | 21.1 (16.1 ,27.1) | 16.5 (9.8 ,26.5) |
| high school | 25.0 (18.3 ,33.1) | 26.7 (22.0 ,32.0) | 28.5 (20.8 ,37.7) | 29.4 (24.0 ,35.3) | 27.6 (20.7 ,35.6) | 28.0 (19.8 ,37.8) | 28.4 (22.7 ,34.9) | 28.0 (23.4 ,33.1) | 35.1 (28.2 ,42.7) |
| >high school | 35.7 (27.7 ,44.5) | 43.7 (35.3 ,52.4) | 42.3 (31.9 ,53.4) | 38.4 (31.3 ,45.9) | 43.0 (34.6 ,51.8) | 46.5 (38.6 ,54.6) | 47.8 (41.5 ,54.3) | 50.9 (43.9 ,57.8) | 48.3 (39.2 ,57.5) |
| Marital status |  |  |  |  |  |  |  |  |  |
| Married/Living with partner | 60.9 (53.4 ,68.0) | 60.9 (50.8 ,70.2) | 57.3 (52.2 ,62.2) | 61.8 (55.5 ,67.8) | 63.7 (57.5 ,69.4) | 60.7 (51.3 ,69.3) | 57.1 (50.5 ,63.4) | 57.3 (51.3 ,63.0) | 60.6 (53.1 ,67.7) |
| Widowed/ Divorced/ Separated | 32.1 (25.1 ,40.2) | 32.9 (24.2 ,42.9) | 39.2 (33.9 ,44.7) | 31.0 (25.0 ,37.7) | 30.9 (25.9 ,36.4) | 32.2 (26.5 ,38.6) | 35.1 (29.5 ,41.1) | 32.6 (27.4 ,38.3) | 34.4 (28.0 ,41.4) |
| Never married | 6.9 (4.2 ,11.2) | 6.2 (3.4 ,11.1) | 3.5 (2.3 ,5.5) | 7.2 (3.9 ,12.7) | 5.4 (3.4 ,8.6) | 7.1 (3.5 ,14.0) | 7.8 (5.2 ,11.6) | 10.1 (7.1 ,14.2) | 5.0 (3.4 ,7.2) |
| PIR |  |  |  |  |  |  |  |  |  |
| <1 | 14.6 (9.7 ,21.4) | 12.8 (9.0 ,18.0) | 15.2 (10.2 ,22.1) | 17.7 (12.5 ,24.6) | 17.5 (12.7 ,23.5) | 19.4 (14.5 ,25.6) | 17.5 (10.7 ,27.4) | 16.4 (10.6 ,24.5) | 13.8 (10.7 ,17.6) |
| 1-2 | 28.4 (21.0 ,37.3) | 32.3 (25.4 ,40.0) | 24.6 (17.8 ,33.0) | 27.4 (24.4 ,30.7) | 26.4 (20.6 ,33.2) | 26.0 (18.6 ,35.1) | 32.2 (24.1 ,41.6) | 30.7 (24.0 ,38.3) | 23.6 (19.5 ,28.4) |
| 2-3 | 16.1 (9.7 ,25.7) | 17.6 (12.9 ,23.5) | 21.5 (15.0 ,30.0) | 16.3 (11.2 ,23.2) | 11.8 (7.8 ,17.4) | 14.8 (8.6 ,24.4) | 16.0 (11.3 ,22.0) | 12.9 (6.9 ,22.9) | 17.8 (13.1 ,23.6) |
| 3-4 | 9.0 (5.6 ,14.2) | 9.5 (4.4 ,19.5) | 12.9 (8.3 ,19.3) | 6.2 (3.9 ,9.7) | 14.7 (11.3 ,19.0) | 8.4 (4.6 ,14.8) | 10.9 (7.0 ,16.8) | 12.1 (6.5 ,21.5) | 6.4 (4.4 ,9.2) |
| 4-5 | 11.3 (6.2 ,19.6) | 9.9 (5.0 ,18.4) | 4.6 (2.5 ,8.2) | 6.2 (3.4 ,11.0) | 6.4 (4.2 ,9.7) | 9.8 (5.3 ,17.2) | 6.7 (3.2 ,13.5) | 7.6 (3.1 ,17.5) | 4.5 (1.9 ,10.3) |
| ≥5 | 15.8 (9.4 ,25.3) | 13.5 (8.6 ,20.4) | 14.7 (9.9 ,21.2) | 17.6 (12.6 ,24.1) | 18.1 (10.9 ,28.6) | 14.4 (9.4 ,21.4) | 12.3 (8.3 ,17.9) | 11.0 (6.7 ,17.5) | 23.3 (16.6 ,31.8) |
| Unknown | 4.8 (2.5 ,8.8) | 4.4 (2.2 ,8.6) | 6.5 (3.3 ,12.4) | 8.5 (6.3 ,11.5) | 5.0 (3.3 ,7.6) | 7.1 (3.8 ,12.9) | 4.4 (2.8 ,6.9) | 9.3 (5.7 ,14.8) | 10.5 (5.7 ,18.6) |
| BMI |  |  |  |  |  |  |  |  |  |
| <25kg/m^2^ | 17.9 (13.9 ,22.8) | 25.9 (19.5 ,33.6) | 17.6 (13.7 ,22.3) | 24.5 (20.3 ,29.3) | 21.5 (18.5 ,24.8) | 22.7 (17.9 ,28.3) | 24.3 (18.9 ,30.6) | 19.7 (15.6 ,24.5) | 16.4 (12.2 ,21.7) |
| 25 to 30kg/m^2^ | 35.2 (25.7 ,45.9) | 36.0 (29.5 ,43.1) | 35.3 (28.9 ,42.3) | 33.8 (29.0 ,39.0) | 28.1 (23.2 ,33.6) | 28.5 (21.4 ,36.8) | 32.0 (26.2 ,38.4) | 29.3 (24.1 ,35.0) | 31.3 (22.6 ,41.6) |
| ≥30kg/m^2^ | 35.1 (25.7 ,45.7) | 35.2 (30.2 ,40.6) | 43.2 (37.2 ,49.4) | 39.0 (33.1 ,45.2) | 48.6 (43.6 ,53.7) | 44.2 (35.4 ,53.4) | 41.2 (32.7 ,50.2) | 48.0 (41.4 ,54.6) | 49.5 (41.4 ,57.5) |
| Unknown | 11.8 (6.2 ,21.2) | 2.8 (1.7 ,4.6) | 3.9 (2.1 ,7.0) | 2.7 (1.8 ,4.1) | 1.8 (0.8 ,3.9) | 4.6 (2.0 ,10.3) | 2.5 (1.3 ,5.0) | 3.1 (1.2 ,7.8) | 2.9 (1.0 ,7.6) |
| Take medication for dyslipidemia |  |  |  |  |  |  |  |  |  |
| Yes | 38.5 (32.7 ,44.6) | 34.8 (28.5 ,41.6) | 42.9 (36.7 ,49.4) | 43.6 (38.1 ,49.2) | 42.1 (35.3 ,49.3) | 61.6 (52.0 ,70.3) | 57.8 (52.5 ,62.9) | 57.4 (47.6 ,66.6) | 58.4 (49.2 ,67.0) |
| No | 5.2 (3.0 ,8.9) | 7.3 (3.4 ,15.3) | 1.8 (0.9 ,3.3) | 7.3 (5.1 ,10.3) | 4.9 (3.0 ,8.1) | 5.6 (3.3 ,9.2) | 5.9 (4.0 ,8.7) | 8.4 (4.5 ,14.9) | 13.0 (9.0 ,18.3) |
| Unknown | 56.3 (48.6 ,63.7) | 57.9 (49.2 ,66.0) | 55.3 (48.7 ,61.8) | 49.2 (43.6 ,54.8) | 52.9 (45.7 ,60.0) | 32.9 (25.1 ,41.7) | 36.3 (31.0 ,42.0) | 34.2 (27.7 ,41.4) | 28.7 (22.5 ,35.7) |
| Smoke |  |  |  |  |  |  |  |  |  |
| Yes | 67.6 (59.4 ,74.8) | 65.8 (57.1 ,73.6) | 68.5 (60.9 ,75.3) | 62.2 (55.3 ,68.6) | 59.7 (53.9 ,65.2) | 63.7 (54.5 ,72.0) | 64.1 (59.0 ,69.0) | 62.8 (53.1 ,71.7) | 60.7 (53.8 ,67.2) |
| No | 32.4 (25.2 ,40.6) | 34.2 (26.4 ,42.9) | 31.5 (24.7 ,39.1) | 37.8 (31.4 ,44.7) | 40.3 (34.8 ,46.1) | 36.3 (28.0 ,45.5) | 35.9 (31.0 ,41.0) | 37.2 (28.3 ,46.9) | 39.3 (32.8 ,46.2) |

Abbreviation: HDL,high-density lipoprotein; PIR, poverty to income ratio; BMI, body mass index

^a^ Continuous variable presented as survey-weighted mean (95% CI).

Categorical variables were presented as survey-weighted percentage (95% CI).

The survey weighting method occasionally produced estimates in decimal numbers. The sum of the numbers may not add up to the heading totals when they are rounded and added.

Table S3 Weighted demographic characteristics of survivors for trend analysis of LDL

| Variables | 2001-2002 | 2003-2004 | 2005-2006 | 2007-2008 | 2009-2010 | 2011-2012 | 2013-2014 | 2015-2016 | 2017-2018 |
| --- | --- | --- | --- | --- | --- | --- | --- | --- | --- |
| Weighted sample size | 3488630 | 4020687 | 5137148 | 5713706 | 4999566 | 6145412 | 6063502 | 5786546 | 6029799 |
| Un-weighted sample size | 131 | 141 | 152 | 221 | 189 | 161 | 173 | 171 | 213 |
| Age,y^a^ | 63.8 (60.7 ,66.9) | 64.4 (61.2 ,67.6) | 64.3 (61.6 ,67.1) | 66.7 (64.1 ,69.4) | 63.2 (60.9 ,65.5) | 65.3 (63.1 ,67.5) | 66.5 (63.6 ,69.4) | 63.7 (62.7 ,64.7) | 64.2 (61.8 ,66.7) |
| Sex |  |  |  |  |  |  |  |  |  |
| Man | 56.7 (47.7 ,65.3) | 57.0 (46.3 ,67.0) | 50.9 (38.8 ,62.9) | 54.1 (45.9 ,62.2) | 52.2 (44.2 ,60.2) | 56.5 (46.7 ,65.9) | 53.4 (42.1 ,64.4) | 58.1 (50.0 ,65.8) | 54.7 (45.9 ,63.2) |
| Women | 43.3 (34.7 ,52.3) | 43.0 (33.0 ,53.7) | 49.1 (37.1 ,61.2) | 45.9 (37.8 ,54.1) | 47.8 (39.8 ,55.8) | 43.5 (34.1 ,53.3) | 46.6 (35.6 ,57.9) | 41.9 (34.2 ,50.0) | 45.3 (36.8 ,54.1) |
| race |  |  |  |  |  |  |  |  |  |
| Non-Hispanic White | 82.6 (72.4 ,89.5) | 78.3 (66.5 ,86.7) | 77.7 (64.1 ,87.1) | 76.4 (67.9 ,83.2) | 74.7 (64.6 ,82.6) | 76.0 (64.3 ,84.7) | 77.6 (67.1 ,85.4) | 63.0 (50.2 ,74.2) | 63.7 (55.2 ,71.5) |
| Other races | 17.4 (10.5 ,27.6) | 21.7 (13.3 ,33.5) | 22.3 (12.9 ,35.9) | 23.6 (16.8 ,32.1) | 25.3 (17.4 ,35.4) | 24.0 (15.3 ,35.7) | 22.4 (14.6 ,32.9) | 37.0 (25.8 ,49.8) | 36.3 (28.5 ,44.8) |
| Educational level |  |  |  |  |  |  |  |  |  |
| <high school | 33.5 (23.0 ,45.8) | 32.2 (23.6 ,42.1) | 27.6 (19.9 ,36.9) | 35.4 (29.1 ,42.3) | 32.3 (25.3 ,40.3) | 22.5 (14.2 ,33.6) | 27.4 (19.7 ,36.7) | 23.8 (16.0 ,33.8) | 21.4 (10.0 ,40.0) |
| high school | 28.9 (17.8 ,43.4) | 25.3 (20.7 ,30.5) | 28.4 (19.4 ,39.5) | 28.6 (21.9 ,36.5) | 27.0 (19.1 ,36.6) | 26.8 (17.6 ,38.5) | 24.3 (16.8 ,33.7) | 29.6 (23.4 ,36.7) | 31.3 (18.6 ,47.6) |
| >high school | 37.6 (27.4 ,49.0) | 42.5 (33.2 ,52.4) | 44.0 (32.0 ,56.8) | 35.9 (26.6 ,46.4) | 40.7 (29.6 ,52.9) | 50.8 (40.8 ,60.6) | 48.3 (40.5 ,56.3) | 46.6 (38.5 ,54.9) | 47.3 (30.4 ,64.9) |
| Marital status |  |  |  |  |  |  |  |  |  |
| Married/Living with partner | 62.7 (49.2 ,74.5) | 64.7 (53.1 ,74.8) | 60.7 (51.5 ,69.2) | 60.0 (51.1 ,68.3) | 64.5 (53.6 ,74.1) | 66.9 (56.7 ,75.8) | 63.4 (56.5 ,69.7) | 61.5 (48.7 ,72.9) | 66.2 (56.9 ,74.5) |
| Widowed/ Divorced/ Separated | 28.8 (18.8 ,41.4) | 30.2 (21.2 ,41.0) | 37.7 (28.8 ,47.5) | 33.8 (24.6 ,44.4) | 29.5 (21.1 ,39.6) | 29.8 (22.0 ,39.0) | 31.7 (25.5 ,38.7) | 29.0 (20.1 ,39.9) | 29.3 (21.7 ,38.3) |
| Never married | 8.5 (3.8 ,17.9) | 5.1 (1.9 ,13.4) | 1.6 (0.6 ,4.3) | 6.2 (2.7 ,13.7) | 6.0 (3.1 ,11.1) | 3.3 (1.6 ,6.7) | 4.9 (2.4 ,9.8) | 9.5 (4.9 ,17.6) | 4.4 (2.7 ,7.3) |
| PIR |  |  |  |  |  |  |  |  |  |
| <1 | 16.6 (9.8 ,26.8) | 7.4 (4.0 ,13.3) | 18.2 (11.3 ,28.1) | 20.0 (15.1 ,26.0) | 16.9 (10.3 ,26.3) | 13.8 (9.7 ,19.3) | 17.4 (8.4 ,32.6) | 15.3 (7.0 ,30.1) | 15.0 (9.9 ,22.0) |
| 1-2 | 21.3 (15.0 ,29.5) | 33.8 (24.0 ,45.2) | 25.5 (16.9 ,36.6) | 27.9 (22.0 ,34.6) | 30.2 (21.9 ,40.0) | 26.4 (16.5 ,39.5) | 25.8 (16.8 ,37.4) | 29.4 (19.1 ,42.3) | 24.6 (19.5 ,30.4) |
| 2-3 | 13.9 (7.7 ,23.8) | 24.4 (15.7 ,35.8) | 21.1 (13.9 ,30.8) | 15.8 (9.0 ,26.5) | 11.3 (6.1 ,19.8) | 12.7 (6.4 ,23.8) | 17.9 (12.1 ,25.7) | 18.3 (10.0 ,31.0) | 17.3 (11.9 ,24.4) |
| 3-4 | 8.6 (2.8 ,23.8) | 7.9 (3.5 ,16.8) | 15.2 (7.7 ,28.0) | 4.6 (2.4 ,8.6) | 13.0 (7.5 ,21.6) | 10.4 (4.6 ,21.9) | 11.4 (6.6 ,19.1) | 12.4 (5.2 ,26.9) | 7.5 (3.0 ,17.3) |
| 4-5 | 12.3 (5.0 ,26.9) | 10.8 (4.9 ,22.0) | 2.6 (0.7 ,8.7) | 6.0 (1.9 ,17.4) | 5.6 (3.1 ,9.9) | 11.7 (5.8 ,22.5) | 10.0 (3.8 ,23.7) | 6.3 (1.6 ,21.7) | 4.1 (1.3 ,12.2) |
| ≥5 | 20.4 (12.0 ,32.6) | 10.4 (5.2 ,19.6) | 13.0 (7.0 ,22.9) | 17.2 (11.4 ,25.1) | 17.3 (8.7 ,31.2) | 15.4 (8.6 ,26.1) | 14.0 (9.0 ,21.2) | 7.1 (2.6 ,18.0) | 20.0 (11.4 ,32.8) |
| Unknown | 6.8 (3.5 ,12.8) | 5.3 (2.2 ,12.0) | 4.2 (1.9 ,9.0) | 8.4 (5.6 ,12.4) | 5.8 (3.2 ,10.3) | 9.5 (5.5 ,15.9) | 3.4 (1.5 ,7.3) | 11.3 (5.1 ,23.3) | 11.6 (4.2 ,28.2) |
| BMI |  |  |  |  |  |  |  |  |  |
| <25kg/m^2^ | 23.4 (17.4 ,30.7) | 21.7 (13.6 ,32.8) | 20.6 (13.7 ,29.9) | 24.4 (18.1 ,32.1) | 23.9 (18.4 ,30.5) | 27.3 (20.4 ,35.6) | 26.5 (20.2 ,34.1) | 20.7 (15.6 ,27.1) | 22.1 (14.3 ,32.4) |
| 25 to 30kg/m^2^ | 37.1 (25.5 ,50.4) | 38.4 (29.3 ,48.4) | 28.7 (21.1 ,37.8) | 31.2 (25.0 ,38.0) | 24.1 (18.9 ,30.1) | 24.5 (16.2 ,35.4) | 32.1 (23.5 ,42.2) | 30.1 (21.8 ,39.9) | 31.6 (21.3 ,44.2) |
| ≥30kg/m^2^ | 29.1 (17.8 ,43.8) | 38.9 (29.4 ,49.4) | 47.8 (36.5 ,59.3) | 41.0 (31.6 ,51.0) | 51.2 (46.0 ,56.3) | 45.9 (33.4 ,59.0) | 40.0 (28.3 ,52.9) | 45.5 (34.3 ,57.3) | 43.5 (32.3 ,55.4) |
| Unknown | 10.4 (4.7 ,21.4) | 1.0 (0.3 ,3.4) | 2.9 (1.2 ,7.1) | 3.5 (1.7 ,6.8) | 0.8 (0.2 ,3.7) | 2.2 (0.7 ,6.6) | 1.4 (0.6 ,3.0) | 3.6 (0.9 ,14.2) | 2.8 (0.9 ,8.3) |
| Take medication for dyslipidemia |  |  |  |  |  |  |  |  |  |
| Yes | 39.8 (34.4 ,45.5) | 36.7 (25.0 ,50.3) | 45.2 (38.7 ,51.8) | 47.1 (38.3 ,56.1) | 42.1 (32.4 ,52.5) | 61.0 (48.9 ,71.9) | 60.4 (51.7 ,68.4) | 61.7 (52.0 ,70.6) | 58.4 (47.7 ,68.3) |
| No | 5.1 (1.9 ,13.3) | 6.5 (2.1 ,18.7) | 2.1 (1.0 ,4.1) | 4.4 (2.3 ,8.1) | 4.6 (2.0 ,10.3) | 4.3 (2.1 ,8.8) | 6.3 (4.3 ,9.2) | 4.8 (1.9 ,11.6) | 12.2 (7.0 ,20.3) |
| Unknown | 55.0 (47.2 ,62.6) | 56.8 (41.3 ,71.1) | 52.7 (47.1 ,58.3) | 48.5 (40.4 ,56.6) | 53.3 (44.2 ,62.1) | 34.7 (23.3 ,48.1) | 33.3 (26.0 ,41.5) | 33.5 (27.3 ,40.3) | 29.4 (21.6 ,38.8) |
| Smoke |  |  |  |  |  |  |  |  |  |
| Yes | 74.8 (63.8 ,83.3) | 59.8 (47.2 ,71.2) | 71.0 (58.9 ,80.7) | 63.8 (52.7 ,73.6) | 58.2 (49.6 ,66.3) | 66.5 (52.9 ,77.8) | 65.4 (55.0 ,74.6) | 66.1 (48.2 ,80.3) | 62.7 (53.8 ,70.9) |
| No | 25.2 (16.7 ,36.2) | 40.2 (28.8 ,52.8) | 29.0 (19.3 ,41.1) | 36.2 (26.4 ,47.3) | 41.8 (33.7 ,50.4) | 33.5 (22.2 ,47.1) | 34.6 (25.4 ,45.0) | 33.9 (19.7 ,51.8) | 37.3 (29.1 ,46.2) |

Abbreviation: LDL,low-density lipoprotein; PIR, poverty to income ratio; BMI, body mass index

^a^ Continuous variable presented as survey-weighted mean (95% CI).

Categorical variables were presented as survey-weighted percentage (95% CI).

The survey weighting method occasionally produced estimates in decimal numbers. The sum of the numbers may not add up to the heading totals when they are rounded and added.

Table S4 Weighted demographic characteristics of survivors for trend analysis of triglyceride

| Variables | 2001-2002 | 2003-2004 | 2005-2006 | 2007-2008 | 2009-2010 | 2011-2012 | 2013-2014 | 2015-2016 | 2017-2018 |
| --- | --- | --- | --- | --- | --- | --- | --- | --- | --- |
| Weighted sample size | 4084565 | 4204922 | 5229432 | 5775639 | 5024501 | 6263281 | 6150882 | 5808486 | 6152444 |
| Un-weighted sample size | 157 | 148 | 154 | 226 | 191 | 166 | 175 | 172 | 216 |
| Age,y^a^ | 63.9 (61.5 ,66.3) | 64.4 (61.3 ,67.5) | 64.4 (61.7 ,67.0) | 66.5 (63.9 ,69.1) | 63.1 (60.9 ,65.4) | 65.3 (63.1 ,67.4) | 66.5 (63.5 ,69.5) | 63.6 (62.5 ,64.6) | 64.1 (61.7 ,66.6) |
| Sex |  |  |  |  |  |  |  |  |  |
| Man | 58.1 (49.4 ,66.3) | 58.8 (48.4 ,68.4) | 50.0 (38.0 ,62.0) | 54.5 (46.3 ,62.5) | 52.5 (44.5 ,60.4) | 56.4 (46.4 ,65.9) | 52.7 (41.2 ,63.9) | 58.2 (50.2 ,65.9) | 54.9 (46.0 ,63.5) |
| Women | 41.9 (33.7 ,50.6) | 41.2 (31.6 ,51.6) | 50.0 (38.0 ,62.0) | 45.5 (37.5 ,53.7) | 47.5 (39.6 ,55.5) | 43.6 (34.1 ,53.6) | 47.3 (36.1 ,58.8) | 41.8 (34.1 ,49.8) | 45.1 (36.5 ,54.0) |
| race |  |  |  |  |  |  |  |  |  |
| Non-Hispanic White | 82.0 (72.3 ,88.9) | 78.5 (67.1 ,86.7) | 78.1 (64.9 ,87.3) | 76.3 (67.9 ,83.1) | 74.3 (64.0 ,82.5) | 76.3 (64.5 ,85.0) | 77.9 (67.1 ,85.9) | 62.8 (50.1 ,73.9) | 63.8 (54.5 ,72.1) |
| Other races | 18.0 (11.1 ,27.7) | 21.5 (13.3 ,32.9) | 21.9 (12.7 ,35.1) | 23.7 (16.9 ,32.1) | 25.7 (17.5 ,36.0) | 23.7 (15.0 ,35.5) | 22.1 (14.1 ,32.9) | 37.2 (26.1 ,49.9) | 36.2 (27.9 ,45.5) |
| Educational level |  |  |  |  |  |  |  |  |  |
| <high school | 35.0 (24.5 ,47.1) | 32.1 (24.2 ,41.2) | 27.1 (19.6 ,36.2) | 35.6 (29.0 ,42.6) | 32.4 (25.5 ,40.2) | 23.2 (14.8 ,34.3) | 27.9 (20.5 ,36.8) | 23.7 (15.9 ,33.7) | 21.0 (9.7 ,39.5) |
| high school | 31.0 (20.0 ,44.8) | 24.3 (19.7 ,29.4) | 28.6 (20.0 ,39.2) | 28.8 (22.0 ,36.7) | 26.8 (19.0 ,36.4) | 26.5 (17.5 ,38.1) | 23.9 (16.4 ,33.5) | 29.5 (23.3 ,36.5) | 30.7 (18.5 ,46.3) |
| >high school | 34.0 (24.7 ,44.8) | 43.6 (35.0 ,52.6) | 44.3 (32.1 ,57.1) | 35.6 (26.4 ,46.1) | 40.7 (29.8 ,52.7) | 50.3 (40.5 ,60.1) | 48.2 (41.1 ,55.3) | 46.8 (38.7 ,55.1) | 48.3 (31.9 ,65.2) |
| Marital status |  |  |  |  |  |  |  |  |  |
| Married/Living with partner | 63.1 (52.3 ,72.7) | 65.6 (54.3 ,75.3) | 59.6 (49.6 ,68.9) | 60.1 (51.4 ,68.2) | 64.7 (54.0 ,74.1) | 66.8 (57.2 ,75.1) | 63.4 (56.8 ,69.5) | 61.7 (49.0 ,73.0) | 66.2 (56.5 ,74.8) |
| Widowed/ Divorced/ Separated | 29.1 (20.7 ,39.1) | 29.5 (20.9 ,39.8) | 38.8 (29.2 ,49.3) | 33.7 (24.8 ,44.0) | 29.4 (21.1 ,39.3) | 30.0 (22.7 ,38.5) | 31.8 (25.5 ,38.8) | 28.9 (20.0 ,39.7) | 29.4 (21.7 ,38.6) |
| Never married | 7.8 (3.7 ,15.9) | 4.9 (1.8 ,12.9) | 1.6 (0.6 ,4.2) | 6.2 (2.7 ,13.6) | 5.9 (3.1 ,11.0) | 3.2 (1.6 ,6.5) | 4.9 (2.4 ,9.6) | 9.4 (4.9 ,17.5) | 4.4 (2.6 ,7.1) |
| PIR |  |  |  |  |  |  |  |  |  |
| <1 | 16.0 (9.5 ,25.7) | 8.3 (4.6 ,14.7) | 17.9 (11.1 ,27.7) | 20.3 (15.0 ,26.8) | 16.8 (10.3 ,26.2) | 14.7 (10.8 ,19.8) | 17.2 (8.2 ,32.4) | 15.2 (7.0 ,30.0) | 14.7 (9.5 ,21.9) |
| 1-2 | 23.1 (15.6 ,32.7) | 33.2 (23.7 ,44.4) | 25.8 (17.2 ,36.9) | 28.1 (22.3 ,34.7) | 30.0 (21.8 ,39.8) | 26.5 (16.9 ,39.0) | 26.8 (17.9 ,38.1) | 29.7 (19.4 ,42.5) | 24.2 (19.2 ,30.0) |
| 2-3 | 14.4 (7.8 ,25.1) | 23.3 (15.1 ,34.2) | 20.8 (13.8 ,30.0) | 15.8 (9.0 ,26.3) | 11.2 (6.1 ,19.7) | 12.5 (6.2 ,23.6) | 17.7 (11.8 ,25.6) | 18.2 (9.9 ,30.9) | 17.5 (12.3 ,24.3) |
| 3-4 | 9.7 (4.7 ,19.2) | 8.8 (4.0 ,18.3) | 15.0 (7.5 ,27.8) | 4.5 (2.3 ,8.5) | 13.0 (7.5 ,21.6) | 10.2 (4.5 ,21.5) | 11.3 (6.6 ,18.7) | 12.4 (5.1 ,26.8) | 7.3 (3.0 ,16.7) |
| 4-5 | 11.4 (4.8 ,24.6) | 10.3 (4.8 ,20.9) | 2.5 (0.7 ,8.5) | 6.0 (1.9 ,17.3) | 5.6 (3.1 ,9.8) | 11.5 (5.7 ,22.1) | 9.9 (3.9 ,23.1) | 6.2 (1.6 ,21.6) | 4.0 (1.2 ,12.2) |
| ≥5 | 18.1 (10.7 ,29.0) | 10.9 (5.9 ,19.3) | 13.8 (7.7 ,23.4) | 17.0 (11.4 ,24.8) | 17.4 (8.9 ,31.4) | 15.3 (8.6 ,25.8) | 13.8 (8.8 ,21.0) | 7.1 (2.6 ,17.9) | 19.6 (11.2 ,32.0) |
| Unknown | 7.4 (3.9 ,13.7) | 5.1 (2.1 ,11.5) | 4.2 (1.9 ,8.8) | 8.3 (5.5 ,12.3) | 6.0 (3.3 ,10.7) | 9.3 (5.4 ,15.5) | 3.4 (1.5 ,7.2) | 11.3 (5.1 ,23.2) | 12.7 (5.2 ,28.0) |
| BMI |  |  |  |  |  |  |  |  |  |
| <25kg/m^2^ | 22.3 (17.1 ,28.4) | 21.7 (13.7 ,32.7) | 20.3 (13.4 ,29.5) | 24.2 (18.0 ,31.8) | 23.8 (18.3 ,30.4) | 26.8 (19.9 ,35.0) | 26.2 (19.8 ,33.7) | 20.7 (15.5 ,27.0) | 21.6 (13.8 ,32.2) |
| 25 to 30kg/m^2^ | 35.9 (24.6 ,49.0) | 38.8 (29.5 ,49.0) | 28.2 (20.6 ,37.3) | 31.1 (24.8 ,38.0) | 24.2 (18.9 ,30.3) | 25.8 (17.1 ,36.9) | 31.7 (23.4 ,41.3) | 30.3 (22.0 ,40.1) | 31.0 (20.8 ,43.5) |
| ≥30kg/m^2^ | 31.5 (21.0 ,44.4) | 38.6 (29.2 ,48.8) | 48.7 (37.4 ,60.2) | 41.3 (31.9 ,51.3) | 51.2 (45.9 ,56.4) | 45.2 (32.8 ,58.3) | 40.8 (29.5 ,53.2) | 45.4 (34.2 ,57.0) | 44.6 (32.7 ,57.2) |
| Unknown | 10.3 (5.0 ,20.1) | 0.9 (0.3 ,3.2) | 2.8 (1.2 ,6.9) | 3.4 (1.7 ,6.8) | 0.8 (0.2 ,3.7) | 2.2 (0.7 ,6.5) | 1.3 (0.6 ,3.0) | 3.6 (0.8 ,14.2) | 2.8 (0.9 ,8.2) |
| Take medication for dyslipidemia |  |  |  |  |  |  |  |  |  |
| Yes | 40.4 (34.4 ,46.6) | 35.9 (24.5 ,49.2) | 46.2 (39.4 ,53.0) | 47.2 (38.3 ,56.4) | 42.4 (32.7 ,52.8) | 61.4 (49.1 ,72.4) | 60.0 (51.5 ,68.0) | 61.5 (51.8 ,70.3) | 57.9 (47.7 ,67.5) |
| No | 5.3 (2.1 ,12.3) | 6.9 (2.3 ,19.0) | 2.0 (1.0 ,4.1) | 4.3 (2.3 ,8.1) | 4.6 (2.0 ,10.3) | 4.6 (2.2 ,9.1) | 6.2 (4.2 ,9.1) | 4.8 (1.9 ,11.6) | 13.3 (8.4 ,20.4) |
| Unknown | 54.4 (45.3 ,63.2) | 57.2 (41.9 ,71.2) | 51.8 (45.9 ,57.7) | 48.4 (40.3 ,56.7) | 53.0 (44.0 ,61.9) | 34.0 (22.7 ,47.5) | 33.7 (26.4 ,41.8) | 33.7 (27.6 ,40.5) | 28.8 (21.2 ,38.0) |
| Smoke |  |  |  |  |  |  |  |  |  |
| Yes | 72.6 (61.1 ,81.7) | 59.8 (47.1 ,71.3) | 70.5 (57.8 ,80.6) | 64.1 (53.1 ,73.8) | 58.4 (49.9 ,66.4) | 66.4 (53.7 ,77.1) | 65.1 (54.9 ,74.0) | 66.2 (48.4 ,80.4) | 62.9 (53.9 ,71.1) |
| No | 27.4 (18.3 ,38.9) | 40.2 (28.7 ,52.9) | 29.5 (19.4 ,42.2) | 35.9 (26.2 ,46.9) | 41.6 (33.6 ,50.1) | 33.6 (22.9 ,46.3) | 34.9 (26.0 ,45.1) | 33.8 (19.6 ,51.6) | 37.1 (28.9 ,46.1) |

Abbreviation: PIR, poverty to income ratio; BMI, body mass index

^a^ Continuous variable presented as survey-weighted mean (95% CI).

Categorical variables were presented as survey-weighted percentage (95% CI).

The survey weighting method occasionally produced estimates in decimal numbers. The sum of the numbers may not add up to the heading totals when they are rounded and added.
